# Supplementary material for: Systematic analysis reveals the prevalence and principles of bypassable gene essentiality
Source: Nat Commun. 2019 Mar 1;10:1002. doi: 10.1038/s41467-019-08928-1 (PMC6397241; doi:10.1038/s41467-019-08928-1)
Supplement: Supplementary file 1 — Supplementary Information [file 41467_2019_8928_MOESM1_ESM.pdf]

Supplementary Information

Systematic analysis reveals the prevalence and principles of bypassable gene essentiality

Jun Li†, Hai-Tao Wang†, Wei-Tao Wang†, Xiao-Ran Zhang, Fang Suo, Jing-Yi Ren, Ying Bi, Ying-Xi Xue, Wen Hu, Meng-Qiu Dong, and Li-Lin Du

†These authors contributed equally to this work.

TABLE OF CONTENTS

Supplementary Figure 1 2
Supplementary Figure 2 4
Supplementary Figure 3 6
Supplementary Figure 4 7
Supplementary Figure 5 8
Supplementary Figure 6 10
Supplementary Figure 7 12
Supplementary Table 1 14
Supplementary Table 2 15

Supplementary Data 1–6 are provided as separate Excel files.

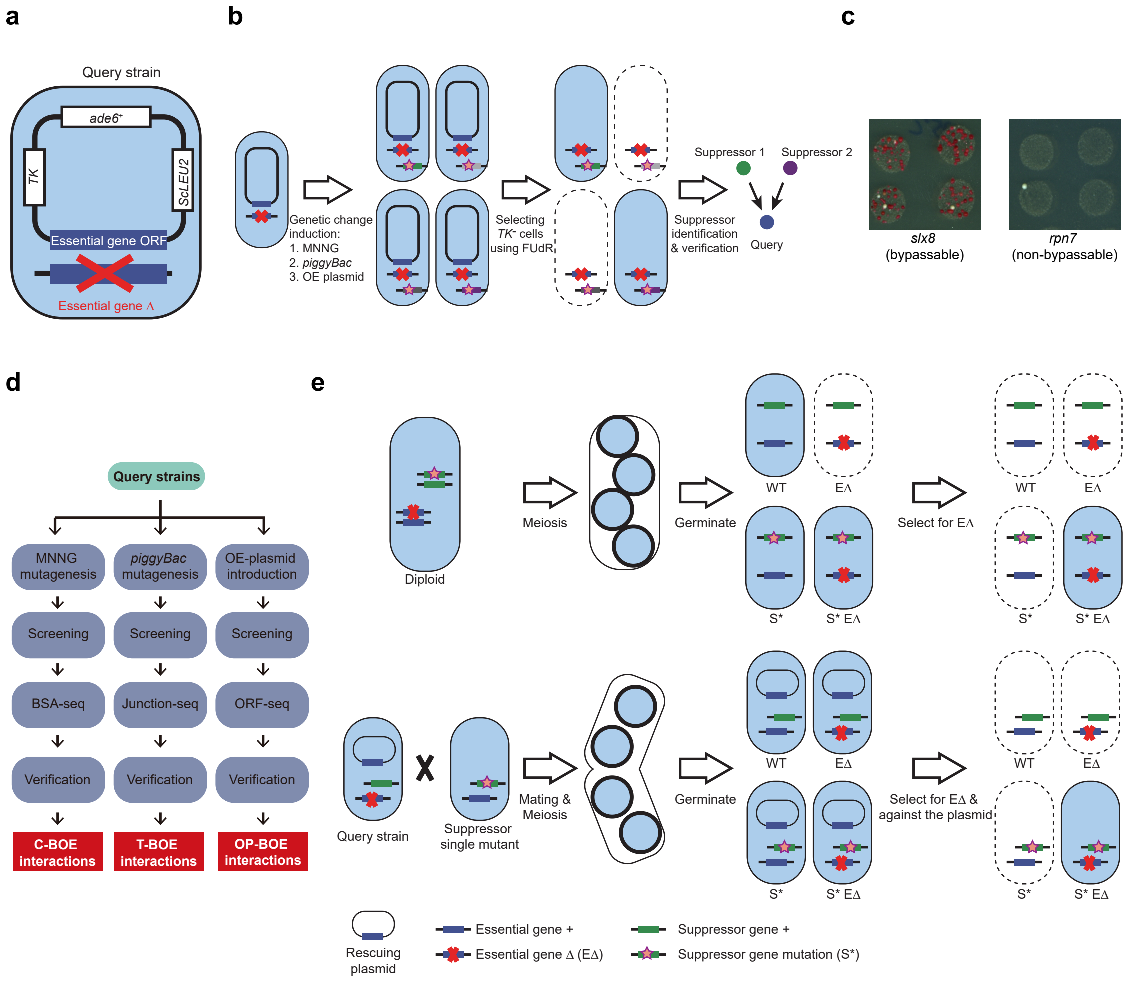

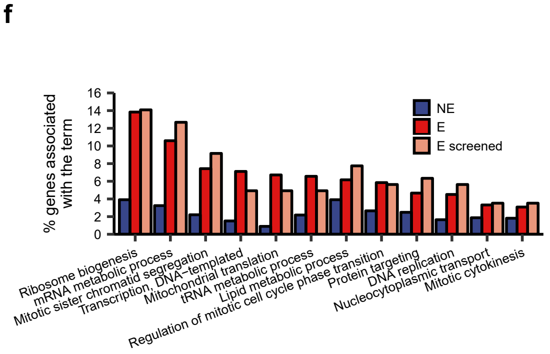

g

Query gene	No. of T-BOE genes	No. of OP-BOE genes	No. of overlapped genes
clr6	12	1	0
cnp20	6	6	0
cwf15	1	3	0
mdm12	2	13	1
mdm35	1	1	0
mmm1	2	12	1
pgr1	11	1	0
pst3	6	2	1
res1	7	6	0
slx8	7	11	1
SPBC27B12.12c	3	1	0
zas1	6	2	2
Total	64	59	6

Supplementary Figure 1. Systematic BOE analysis.

a Schematic of the special strains (termed query strains) constructed for BOE analysis. In each query strain, the chromosomal copy of an essential gene (termed query gene) is deleted, and cell viability is maintained by an episomal plasmid (termed rescuing

plasmid) containing that essential gene and three markers including the counter-selectable thymidine kinase gene (*TK*) and the colony color marker *ade6*$^{+}$.

b Schematic of the BOE analysis. After inducing genetic changes, cells that can lose the rescuing plasmid were selected using 5-fluorodeoxyuridine (FUdR), which kills cells with a functional *TK* gene.

c Representative images of FUdR-containing plates on which suppressor-containing clones were selected. MNNG-mutagenized cells in patches were replica-plated onto YE plates containing FUdR and a low level of adenine (Ade^{-} colonies turn red on plates with a low level of adenine). The white colonies, which still harbored a functional *ade6*$^{+}$ gene, probably resulted from mutational inactivation of the *TK* gene, whereas the red colonies, which were *TK*$^{-}$ and Ade^{-}, probably resulted from a BOE suppressor mutation.

d Flowcharts of the BOE analysis pipelines for the three types of genetic change inducers. Candidate suppressors were identified by deep-sequencing-based methods termed BSA-seq, junction-seq, and ORF-seq, respectively, for C-BOE, T-BOE, and OP-BOE (see Methods for details). We experimentally verified all of the candidate suppressors by independently generating genetic alterations identical or similar to the ones found in the screen hits. Specifically, T-BOE suppressors were verified by deleting the transposon-inserted gene, or by inserting a marker gene at the position of transposon insertion. OP-BOE suppressors were verified by re-introducing the candidate plasmids. C-BOE suppressors were verified by re-creating the candidate mutations.

e Illustration of two verification procedures. In the first procedure, the viability of haploid progenies derived from a diploid heterozygous for both an essential gene deletion and a candidate suppressor was analyzed. Only a real suppressor could allow progenies without the essential gene to form colonies. In the second procedure, the query strain was mated with a strain with a candidate suppressor. Among the haploid progenies with the essential gene deletion, only those with a real suppressor could form colonies in the absence of the rescuing plasmid. The experimental data that verify BOE interactions listed in Supplementary Table S2 can be accessed at https://bypass-of-essentiality.github.io/.

f The GO slim term associations of the 142 query genes ("E screened" in orange) are similar to those of all essential genes ("E" in red) and are different from those of all non-essential genes ("NE" in blue). The 12 GO slim terms most overrepresented among essential genes are shown.

g T-BOE suppressor genes and OP-BOE suppressor genes do not substantially overlap in gene identity. For each of the 12 query essential genes that have both T-BOE and OP-BOE suppressors, the T-BOE suppressor genes were compared to the OP-BOE suppressor genes, and overlapped genes were tallied.

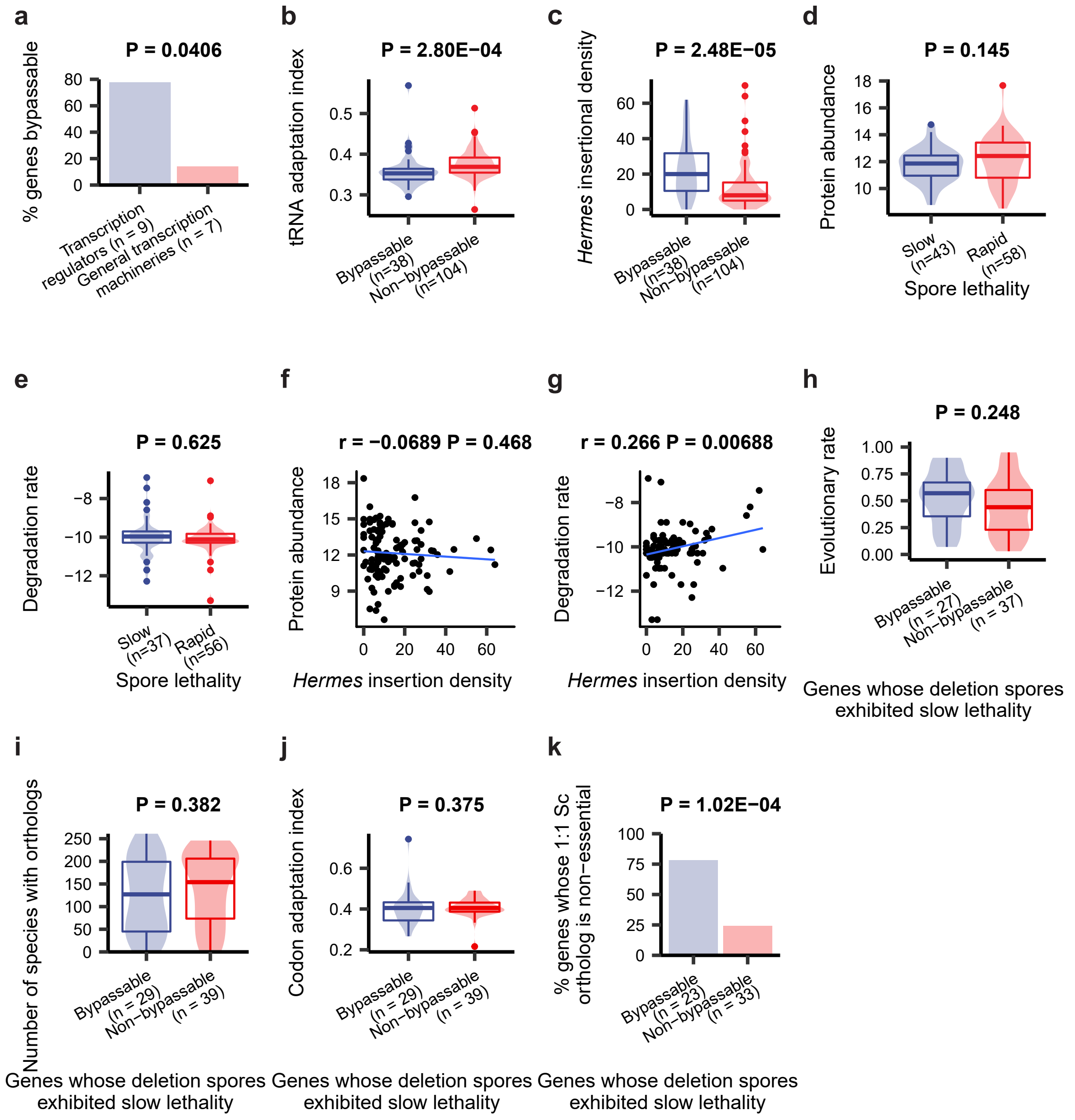

Supplementary Figure 2. Bypassability is correlated with gene importance and differential essentiality between species.

a Genes encoding transcriptional regulators are more likely to be bypassable than are genes encoding parts of general transcription machineries. The P value was calculated using the Fisher's exact test.

b tRNA adaptation indices (tAI) of bypassable and non-bypassable genes. The P value was calculated using the Mann-Whitney-Wilcoxon test.

c *Hermes* insertion densities in bypassable and non-bypassable genes. The P value was calculated using the Mann-Whitney-Wilcoxon test.

d, e Slow spore lethality is not associated with high protein abundance (**d**) or with slow turnover rates (**e**) (only chrII-L query genes were included in the plots; the same conclusions were reached when all essential genes were plotted). P values were calculated using the Mann-Whitney-Wilcoxon test.

f, g High *Hermes* insertion densities are not associated with high protein abundance (**f**) or with slow turnover rates (**g**) (only chrII-L query genes were included in the plots; the same conclusions were reached when all essential genes were plotted). P and r values were calculated using the Pearson correlation analysis.

h-j Evolutionary rates (**h**), the number of species harboring orthologs (**i**), and codon adaptation index (**j**) of bypassable and non-bypassable genes among the query genes with slow spore lethality. P values were calculated using the Mann-Whitney-Wilcoxon test.

k Percentages of slow spore lethality genes whose one-to-one ortholog in *S. cerevisiae* is non-essential. The P value was calculated using the Fisher's exact test.

Boxplots show median (centerline), interquartile range (box), and most extreme data points no further than 1.5-fold interquartile range from either end of the box (whiskers).

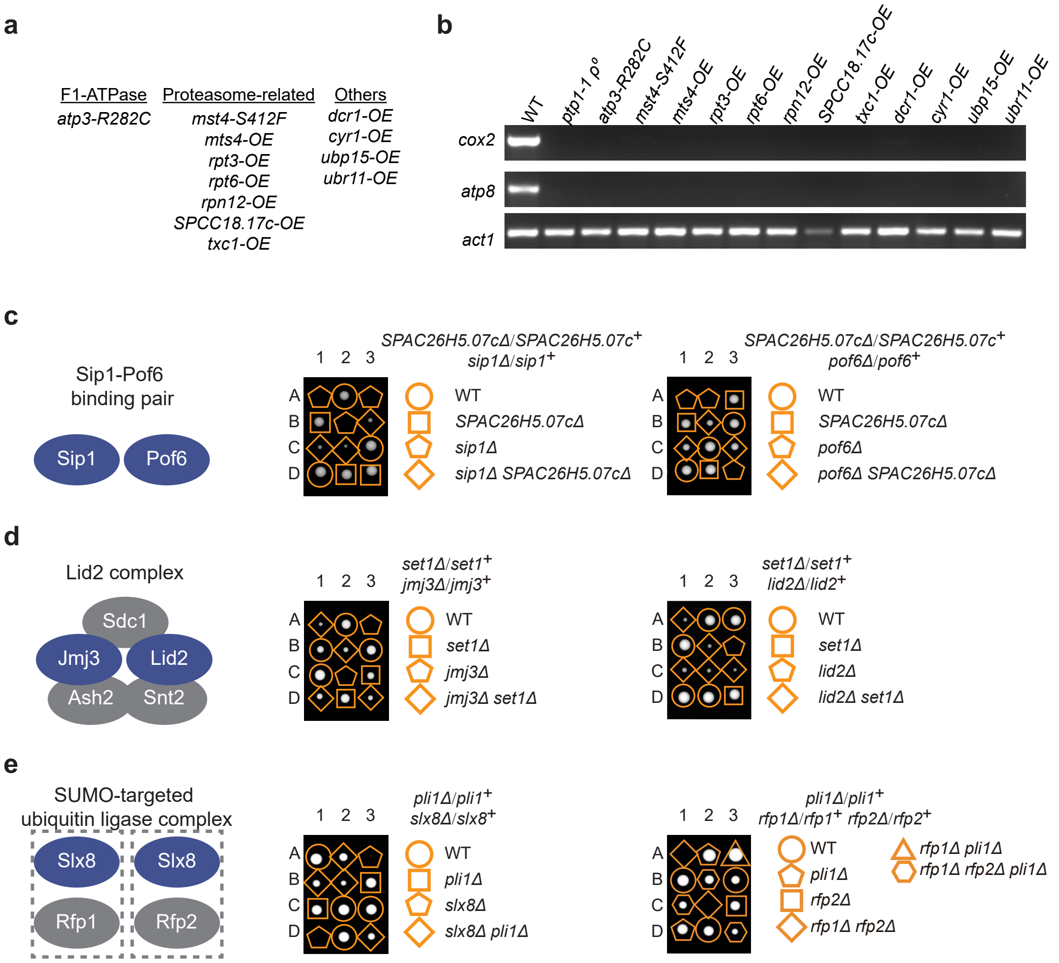

Supplementary Figure 3. Bypassability tends to be shared by genes belonging to the same functional module.

a Classification of the 12 BOE suppressors of mtDNA-expression genes identified in our systematic BOE analysis.

b mtDNA loss was induced by ethidium bromide in strains harboring BOE suppressors of mtDNA-expression genes but not in a wild-type (WT) strain. PCR analysis was performed on ethidium bromide-treated cells. *cox2* and *atp8* are mtDNA-encoded genes. *act1* is a nuclear-encoded gene. A strain known to lack mtDNA (*ptp1-1 ρ*^{0}) was used as a control.

c-e Subunits of the same complex share bypassability and bypass suppressors. *sip1* (**c**), *jmj3* (**d**), and *slx8* (**e**) are three chrII-L-located bypassable essential genes and they encode proteins belonging to three different protein complexes. We asked whether their bypass suppressors can rescue the lethality caused by the deletion of genes encoding other essential subunits of the same complexes. In all three cases, the answer was yes. In (**e**), *rfp1* and *rfp2* encode redundant paralogs that bind to Slx8 and their deletions are synthetic lethal.

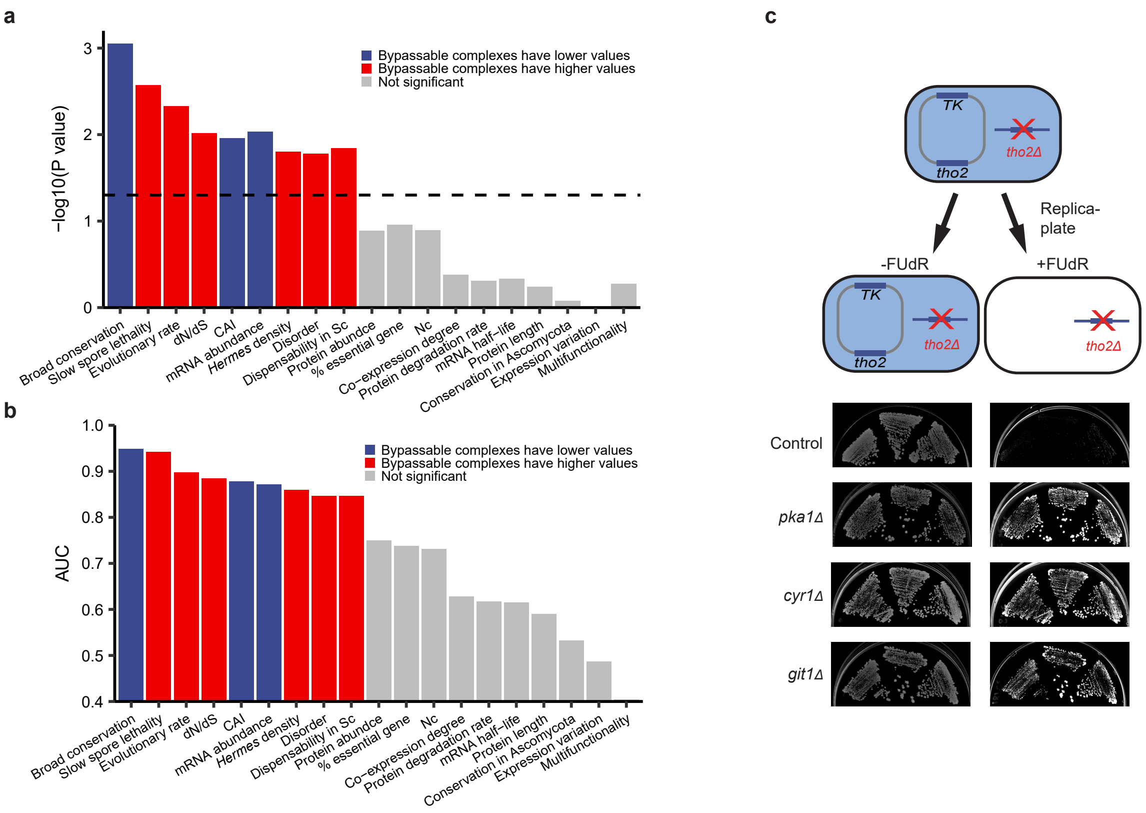

Supplementary Figure 4. Complex bypassability can be predicted.

a 19 quantitative features of essential protein complexes were evaluated for their correlation with protein complex bypassability using the Wilcoxon matched-pairs signed-rank test (bypassable and non-bypassable complexes but not mixed complexes were used in this analysis). Dashed line represents P value = 0.05. The 9 features significantly correlated with bypassability ($P < 0.05$) are highlighted in blue or red according to the relationship between the value of a feature and bypassability. Complex features were calculated as the medians of numeric features of the essential subunits of the complexes or the percentages of categorical features of the essential subunits of the complexes.

b 19 quantitative features of essential protein complexes were evaluated for their ability to predict protein complex bypassability by calculating the area under a ROC curve (AUC) (bypassable and non-bypassable complexes but not mixed complexes were used in this analysis).

c Three BOE suppressors of *tho2*.

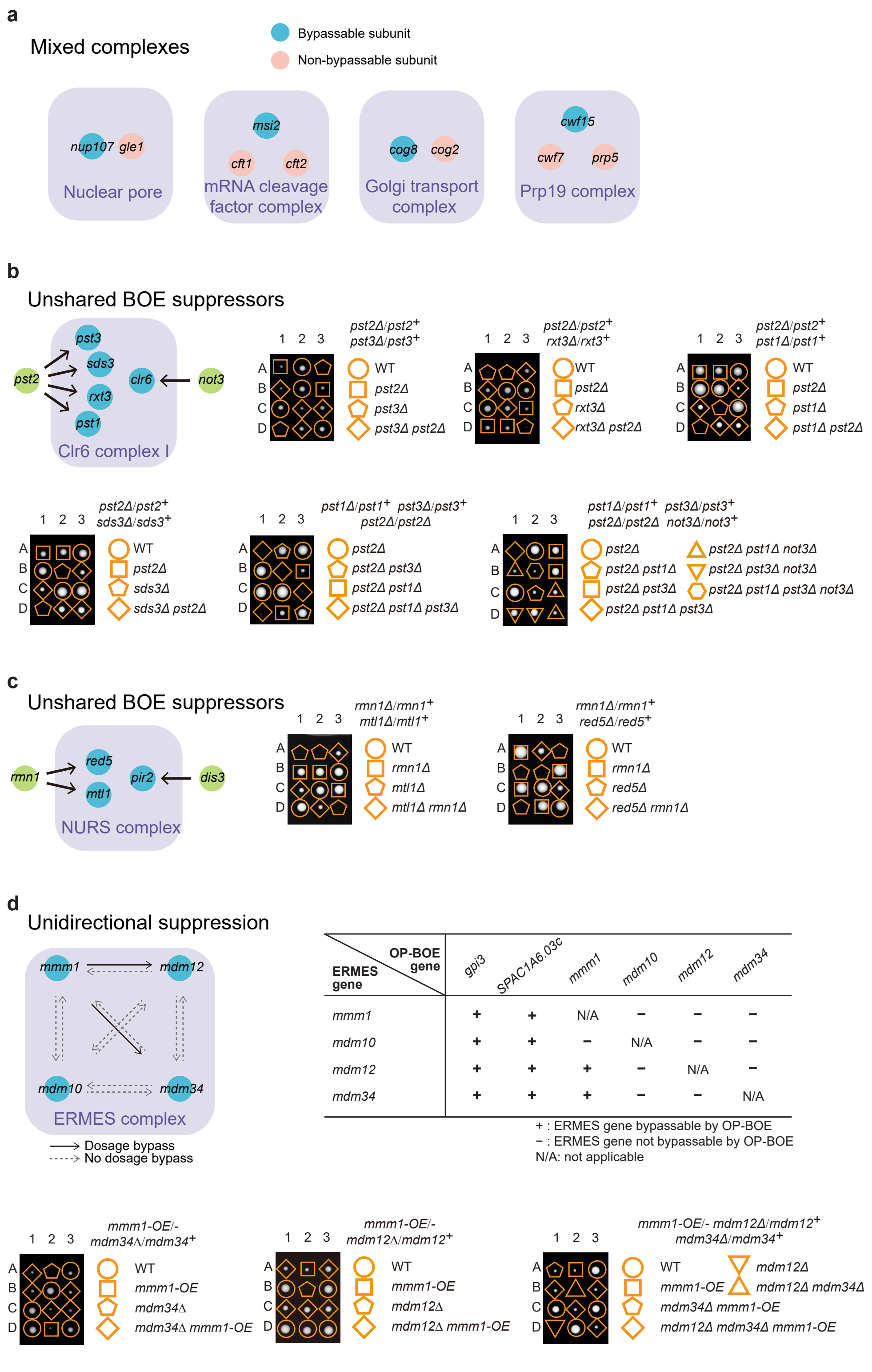

Supplementary Figure 5. Non-uniformity between subunits of the same complex.

a The four examples of "mixed complexes".

b Complex relationships between subunits of Clr6 complexes revealed by BOE analysis.
c Differences within the NURS complex revealed by BOE analysis.
d Differences within the ERMES complex revealed by BOE analysis.

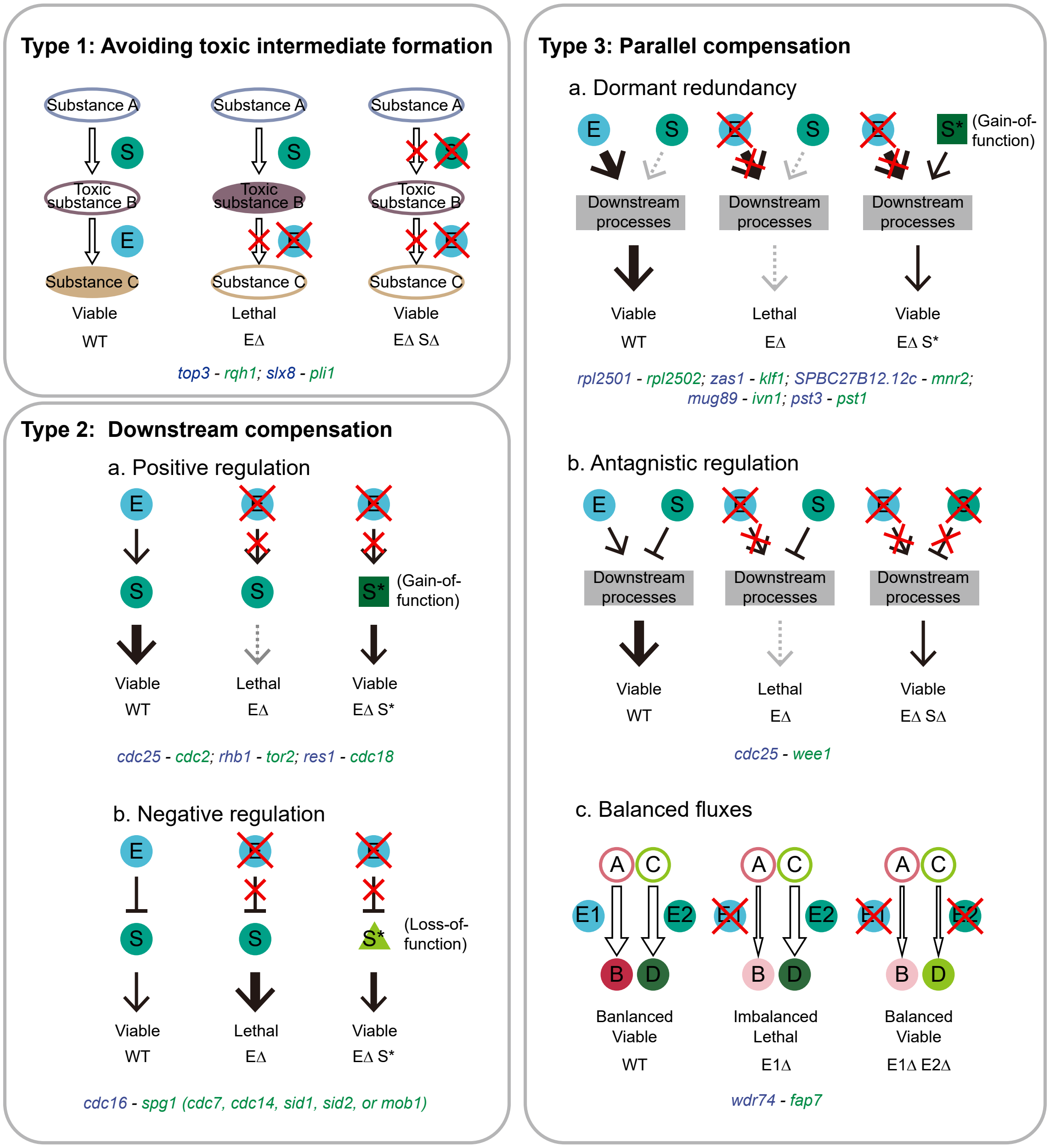

Supplementary Figure 6. Classification of essentiality-bypassing mechanisms for the explainable cases of BOE interactions in fission yeast.

The type 1 mechanism, "bypass by avoiding toxic intermediate formation", describes situations where the loss of an essential gene causes the toxic accumulation of a substance(s) and a BOE suppressor prevents the formation of that substance. The type 2 mechanism, "bypass by downstream compensation", refers to linear signal transduction pathways in which the loss of an essential gene can be compensated for by modulating downstream genes. The type 3 mechanism, "bypass by parallel compensation", describes scenarios in which the loss of an essential gene is compensated for by either increasing a redundant activity or by decreasing a counteracting activity. Cases from fission yeast are given under the schematics. One case of the type 1 mechanism is the bypass of the topoisomerase III gene *top3* by the deletion of the RecQ helicase gene *rqh1*. Rqh1 acts upstream of Top3 biochemically and the DNA structure generated by Rqh1 can kill the cell if not processed by Top3. Another case of the type 1 mechanism is the bypass of the SUMO-targeted ubiquitin ligase (STUbL) gene *slx8* by the deletion of SUMO ligase gene *pli1*. Cases of the type 2 mechanism include the bypass of Cdc25, the activator of cyclin-dependent kinase (CDK), by *cdc2-3w*, a dominant mutation of CDK, the bypass of the Tor2 activator Rhb1 by gain-of-function mutations of *tor2*, the bypass of the transcription factor Res1 by the overexpression of its transcription target gene *cdc18*, and the bypass of *cdc16*, which encodes a negative regulator of the septation initiation network (SIN) pathway by loss-of-function mutations affecting any of the 6 downstream SIN pathway genes. Cases of the type 3 mechanism include the bypass of the 5 chrII-L query genes *rpl2501*, *zas1*, *SPBC27B12.12c*, *mug89*, and *pst3* by overexpressing a paralog, the bypass of *cdc25* by a loss-of-function mutation of *wee1*, which encodes a CDK inhibitor, and the mutual bypass when *wdr74*, an essential gene involved in the assembly of the large ribosomal subunit, and *fap7*, an essential gene involved in the assembly of the small ribosomal subunit, were both deleted.

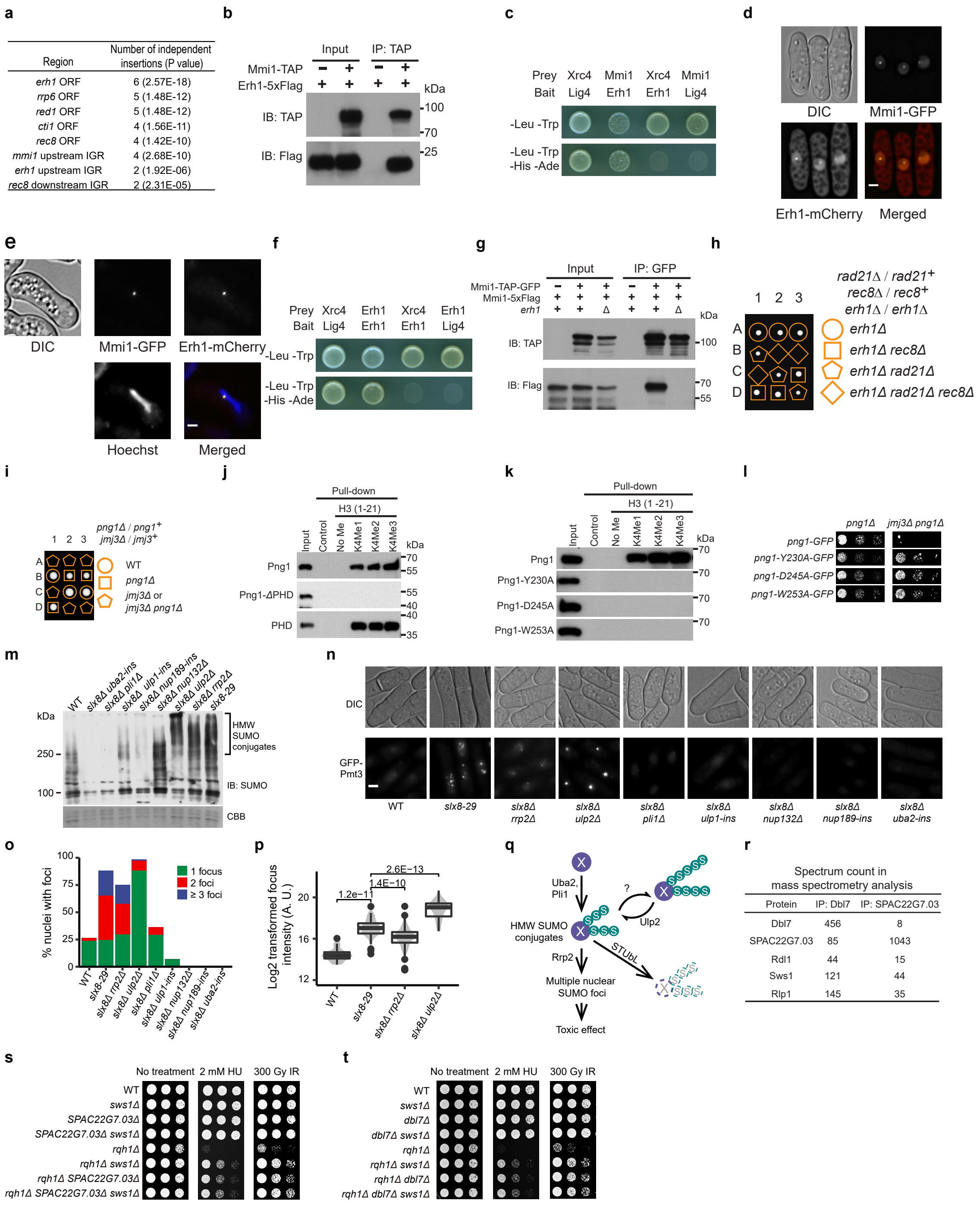

Supplementary Figure 7. Applying BOE analysis to infer gene function.

a Top hits of the *rad21* T-BOE screens. Insertions falling into ORFs and intergenic regions (IGRs) were tallied and the enrichment levels were assessed using P values calculated with G tests.

b Co-immunoprecipitation of Erh1 with Mmi1. TAP-tagged Mmi1 was immunoprecipitated using IgG Sepharose beads. Total cell lysates and IgG precipitates were analyzed by immunoblotting.

c Erh1 and Mmi1 interacted in a yeast two-hybrid assay. The Xrc1-Lig4 interaction served as a positive control. Xrc1 and Lig4 also served as specificity controls.

d Localization of Mmi1 and Erh1 in mitotic cells. Bar, 2 μm.

e Localization of Mmi1 and Erh1 in a cell undergoing meiosis. Bar, 2 μm.

f Erh1 self-interacted in a yeast two-hybrid assay.

g Mmi1-5xFlag co-immunoprecipitated with Mmi1-TAP-GFP in an *erh1*-dependent manner.

h Triple mutant analysis showing that *erh1*Δ bypasses *rad21* in a *rec8*-dependent manner.

i *png1Δ* could not bypass *jmj3*.

j Pull-down of recombinant Png1 by K4-methylated H3 peptides.

k Y230A, D245A, or W253A mutation in Png1 abolished its physical interaction with K4-methylated H3 peptides.

l Y230A, D245A, or W253A mutated version of Png1, but not WT Png1, when expressed, permitted the growth of *jmj3*Δ *png1*Δ.

m Immunoblotting analysis of SUMO conjugates. Coomassie brilliant blue (CBB) staining of membrane served as control for loading and transfer efficiency. Most BOE suppressors of *slx8*, when combined with *slx8Δ*, resulted in lower levels of high-molecular-weight (HMW) SUMO conjugates than the *slx8-29* temperature sensitive mutant at the restrictive temperature. *ulp2Δ* and *rrp2Δ* were two exceptions. The increase of HMW SUMO conjugates in *slx8Δ ulp2Δ* can be explained by the known role of Ulp2 as a SUMO-chain-trimming enzyme.

n Live-cell imaging analysis of the subcellular localization of GFP tagged Pmt3 (SUMO). GFP-Pmt3 formed conspicuous nuclear foci in the *slx8-29* mutant. In comparison, GFP-Pmt3 foci were less bright in *slx8Δ rrp2Δ* but were brighter in *slx8Δ ulp2Δ*. Bar, 2 μm.

o Quantitation of GFP-Pmt3 foci numbers.

p Quantitation of GFP-Pmt3 foci intensity. Boxplots show median (centerline), interquartile range (box), and most extreme data points no further than 1.5-fold interquartile range from either end of the box (whiskers).

q A model depicting the possible roles of several STUbL suppressor genes.

r Mass spectrometry analysis of Dbl7 and SPAC22G7.03 immunoprecipitates.

s *SPAC22G7.03Δ* rescued the DNA damage sensitivity of *rqh1Δ* to the same extent as *sws1Δ* and the rescuing effect was not enhanced when *SPAC22G7.03* and *sws1* were both deleted.

t *dbl7Δ* rescued the DNA damage sensitivity of *rqh1Δ* to the same extent as *sws1Δ* and the rescuing effect was not enhanced when *dbl7* and *sws1* were both deleted.

Source data are provided as a Source Data file.

Supplementary Table 1. Query genes that have paralogs (related to Fig. 5a).

Query gene	Paralog gene(s)	Whether paralog overexpression can bypass the query gene
rpl2501	*rpl2502*	Yes
zas1	*klf1*	Yes
vrs1	*vrs2*	No
pst3	*pst1, pst2*	*pst1* but not *pst2* overexpression can bypass
mug89	*ivn1*	Yes
tor2	*tor1*	No
SPBC27B12.12c	*mnr2*	Yes
cdc13	*cig2*	No
kms2	*kms1*	No

Supplementary Table 2. Primers used in this study.

Name	Sequence (5' to 3')
oligo-128	CCTCACGGGAGCTCCAAGCGGCGAC
adaptor-A	p-GATCGGAAGAGCGGTTCAGCAGGAATGCCGAG-NH_2
adaptor-B	ACCCTTTCTCAGCACATACCGCTCTTCCGATCNNNNNN-NH_2
oligo-498	CAAGCAGAAGACGGCATACGAGATCGGTCTCGGCATTCCTGC TGAACC
Junction-seq-indexed-primer (with index XXXX)	CACGACGCTCTTCCGATCTXXXXACGCAGACTATCTTTCTA
seq-f	AATGATACGGCGACCACCGAGATCTACACTCTTTCCCTACACG ACGCTCTTCCGATCT
seq-r	CAAGCAGAAGACGGCATACGA
ORFseq-up-f (with index XXXX)	CACGACGCTCTTCCGATCTXXXXTACAAAAAAGCAGGCTCT
YFP-5-3	CCACCCCGGTGAACAGCTCCTCGCC
ORFseq-dn-r (with index XXXX)	CACGACGCTCTTCCGATCTXXXXTTTGTACAAGAAAGCTGG
LD214	CCTGGCATATCATCAATTG
t1264_atp8_up	TTAAAAGGTCAGAGTGCAGACTTG
t1265_atp8_dn	TCAATACTTTTGGAGGGGTTAAAT
t1268_cox2_up2	GTTCAATTGTTGAATTCATTTGGA
t1269_cox2_dn2	CACCTTGAACAACAATAGGCATAG
t1270_act1_up2	CATCACACTTTCTACAACGAGCTT
t1271_act1_dn2	TCGTTTCCGATAGTGATAACTTGA
