# Supplementary material for: Systematic analysis reveals the prevalence and principles of bypassable gene essentiality
Source: Nat Commun. 2019 Mar 1;10:1002. doi: 10.1038/s41467-019-08928-1 (PMC6397241; doi:10.1038/s41467-019-08928-1)
Supplement: Supplementary file 3 — Description of Additional Supplementary Files [file 41467_2019_8928_MOESM3_ESM.pdf]

Description of Additional Supplementary Files

File Name: Supplementary Data 1
Description: Essential genes on chrII-L.

File Name: Supplementary Data 2
Description: BOE interactions that bypass the 38 chrII-L essential genes.

File Name: Supplementary Data 3
Description: Essential protein complexes (related to Fig. 4a).

File Name: Supplementary Data 4
Description: Complex features correlated with bypassability (related to Fig. 4b).

File Name: Supplementary Data 5
Description: RNA-seq data.

File Name: Supplementary Data 6
Description: Fission yeast strains used in the follow-up analyses.
